# Elastofibroma Dorsi Presenting as a Subscapular Mass: A Case Report

**DOI:** 10.7759/cureus.111086

**Published:** 2026-06-18

**Authors:** Angie Katherine Osorio, Jesus A Vazquez Calderon, Maria Paula Rangel, Daniela Garcia Ortiz

**Affiliations:** 1 Surgery, Universidad Autónoma de Coahuila, Torreon, MEX; 2 Epidemiology, Fundación Universitaria de Ciencias de la Salud, Bogota, COL

**Keywords:** benign neoplasm, biopsy, elastofibroma, operative procedures, soft tissue neoplasms

## Abstract

Elastofibroma dorsi is a benign tumor that can be clinically misidentified as a malignant neoplasm. Due to its low incidence, few studies to date have focused on determining a standardized surgical approach. We report the case of a female patient with a histopathological diagnosis of elastofibroma dorsi who was managed with surgical excisional biopsy and closed suction drain placement. Postoperative follow-up was conducted over two months to monitor her clinical evolution. The clinical approach implemented at our institution is discussed in comparison with current scientific literature, with the purpose of encouraging further research and awareness of this pathology.

## Introduction

Elastofibroma dorsi is a benign tumor categorized under the fibroblastic/myofibroblastic group according to the 2020 World Health Organization (WHO) classification of soft tissue and bone tumors [1]. Currently, comprehensive research regarding its pathogenesis is limited, and there is no definitive consensus on its standard management. This entity predominantly affects women over 50 years of age, as well as young men; its most common presentation is unilateral [2], although bilateral involvement occurs in approximately 25% of cases. Characteristically, it arises deep to the rhomboid major and latissimus dorsi muscles, adjacent to the inferior angle of the scapula [3]. Awareness of this tumor is crucial because its clinical and imaging presentation mimics malignant lesions, particularly sarcomas, since the mass is often firmly adherent to the chest wall [4]. Clinically, it presents as a palpable mass, with 45% to 54% of patients experiencing symptoms such as pain, shoulder range-of-motion restriction, or paresthesia. Regarding diagnosis, ultrasound, computed tomography (CT), and magnetic resonance imaging (MRI) serve as essential complementary studies, with MRI being the gold standard and documentation via histopathology providing the definitive diagnosis. Several authors note that ultrasound is a highly useful diagnostic tool in resource-limited settings, typically showing an alternating, fascicular, or laminar pattern of hypoechoic and hyperechoic lines parallel to the thoracic wall. CT scans demonstrate alternating areas of adipose tissue (fat density) and fibrous tissue (muscle density) that do not enhance after contrast administration. MRI reveals an alternating pattern of adipose and fibrous tissues; on T1- and T2-weighted sequences, the fibrous tissue yields low-intensity signals similar to muscle, whereas the adipose tissue shows high intensity on T1 and intermediate intensity on T2. On STIR sequences, the lesion appears as areas of mixed high and low intensity that enhance with gadolinium contrast [5]. Regarding treatment, the sparse literature available heavily recommends complete surgical excision of the lesion.

In this study, we present the case of a middle-aged female patient admitted to the Hospital General Universitario Dr. Joaquín del Valle Sánchez in Torreón, Coahuila, Mexico, with a diagnosis of right scapular elastofibroma that required surgical intervention.

## Case presentation

**Figure 1:**
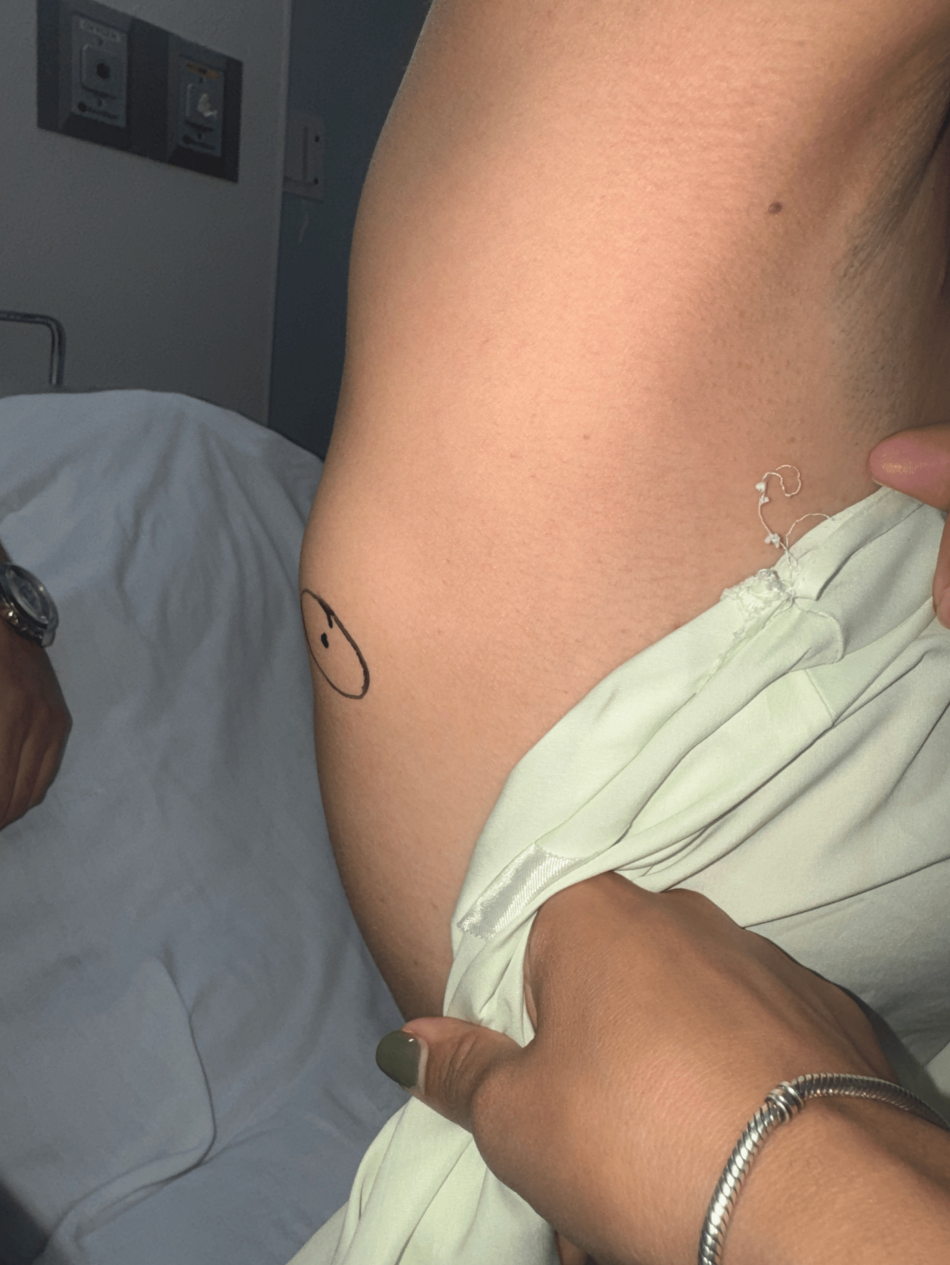
Preoperative clinical view of the right subscapular lesion A physical examination revealed a mass in the right dorsal region with moderately defined borders, limited mobility, and a suspected deep location.

**Figure 2:**
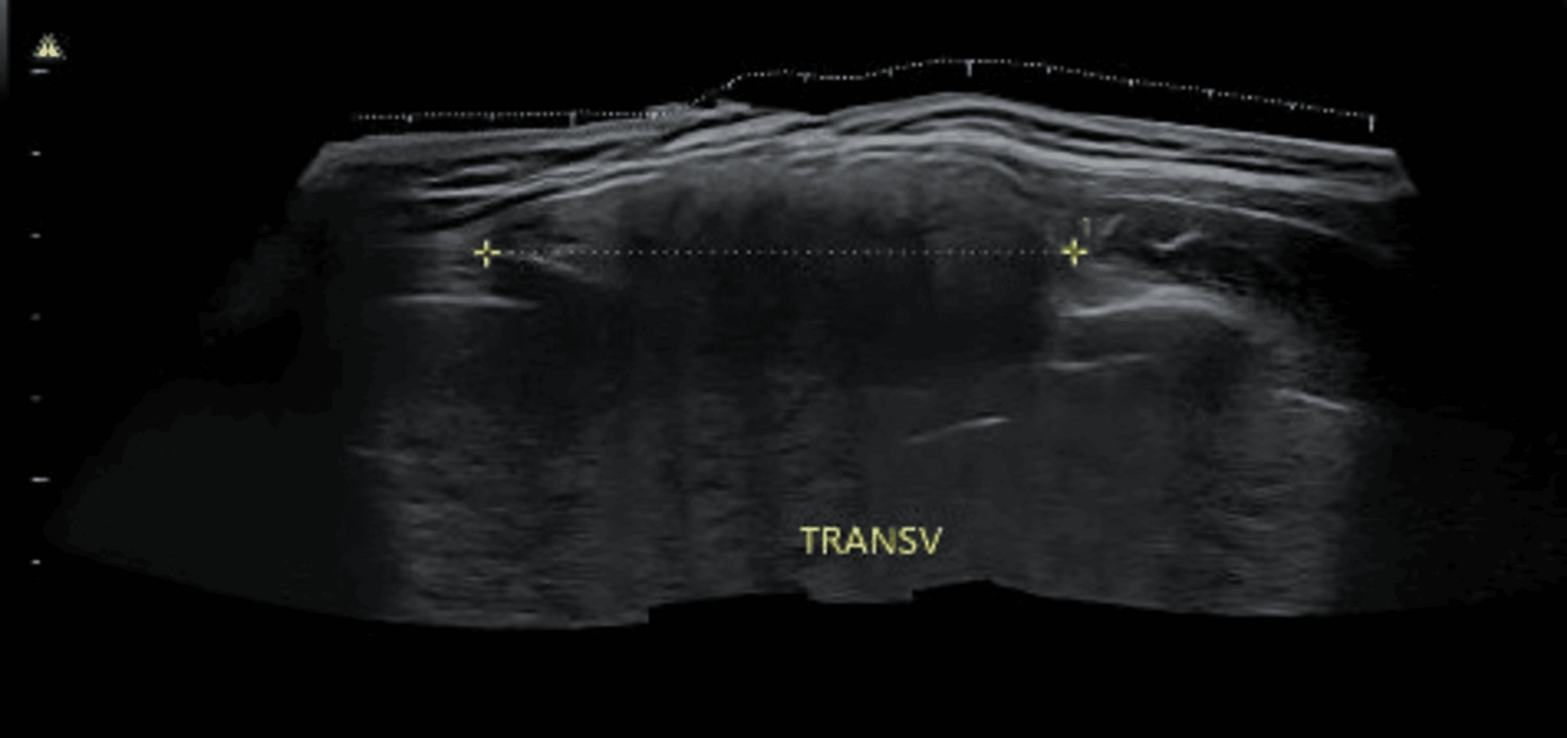
Ultrasound image of the elastofibroma This study revealed a lesion deep to the muscular planes, characterized by an irregular morphology, partially defined margins, and alternating hyperechoic and hypoechoic linear tracts without Doppler vascularity, measuring approximately 5.2 × 7.5 cm. These findings were highly suggestive of elastofibroma dorsi versus sarcoma.

**Figure 3:**
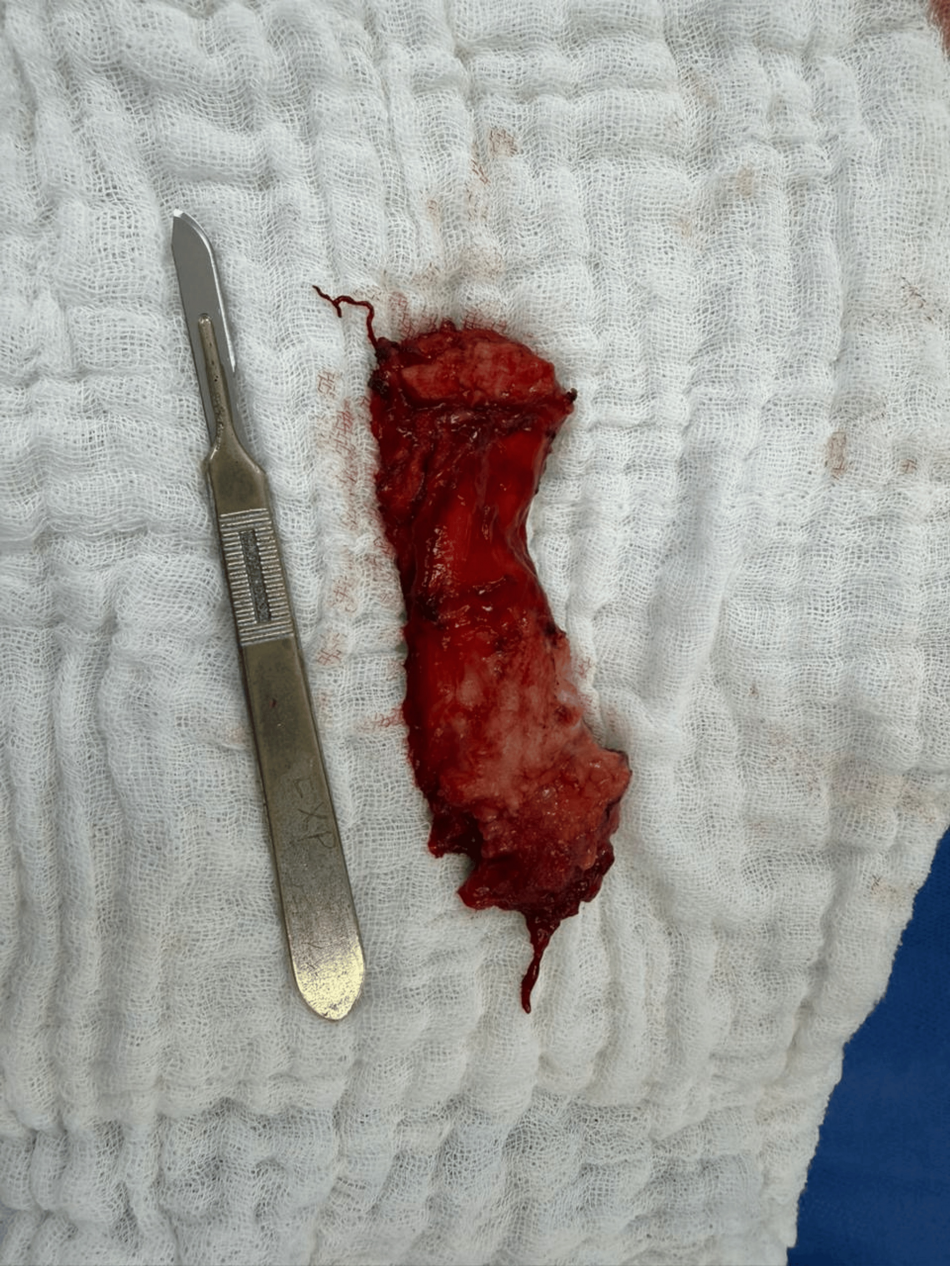
Surgically resected mass A well-defined mass measuring approximately 9 × 4 × 1.5 cm was identified and completely excised from the deep planes without intraoperative complications.

**Figure 4:**
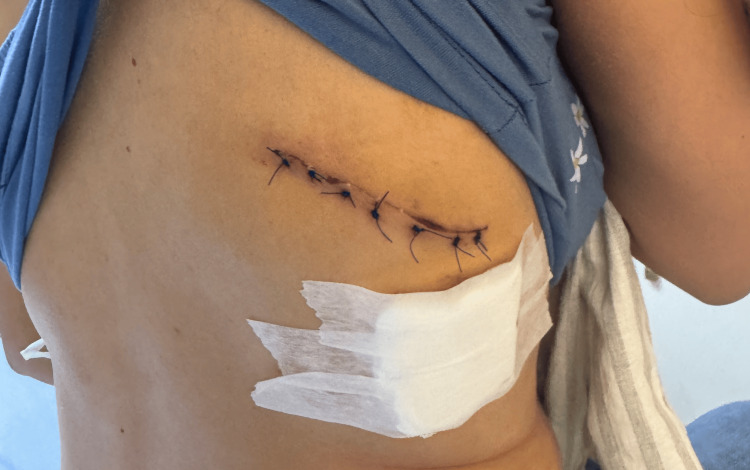
Postoperative view of the sutured subscapular incision A subscapular surgical wound closed with simple sutures without complications; below, the placement site of a drain, which was removed without complications, is evident.

**Figure 5:**
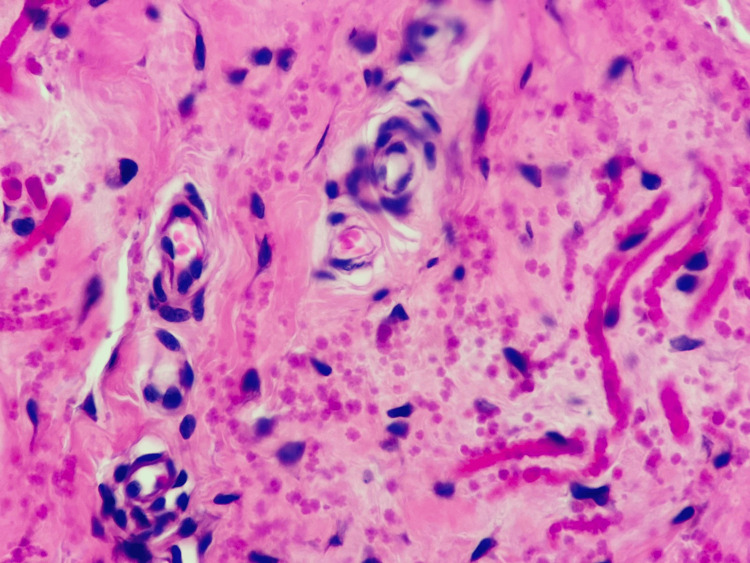
Microscopic histopathology showing characteristic elastic fibers The lesion is composed of dense fibrous connective tissue interspersed with mature adipose tissue. Thick, eosinophilic, fragmented, and disorganized elastic fibers were identified presenting as elongated or globular structures.

A 45-year-old female patient with no relevant medical history, no chronic diseases, no previous surgeries, no allergies, and no regular medication use presented to the general surgery outpatient clinic at Hospital General Universitario Dr. Joaquín del Valle Sánchez in Torreón, Coahuila, Mexico. She reported a three-year history of a gradually increasing mass in the right dorsal region. Over the past four months, she developed pain in the right scapular region during movements of the ipsilateral upper extremity, without functional limitation or paresthesia interfering with her daily activities. Physical examination revealed a mass in the right dorsal region (Figure 1) with moderately defined borders, limited mobility, and a suspected deep location. Routine laboratory investigations were within normal limits. Operating within a resource-limited hospital setting, a soft-tissue ultrasound was requested (Figure 2); this study revealed a lesion deep to the muscular planes, characterized by an irregular morphology, partially defined margins, and alternating hyperechoic and hypoechoic linear tracts without Doppler vascularity, measuring approximately 5.2 × 7.5 cm. These findings were highly suggestive of elastofibroma dorsi versus sarcoma. Consequently, a surgical excisional biopsy was scheduled. Under local anesthesia and intravenous sedation, a transverse incision was made over the tumor in the right dorsal region. A well-defined mass measuring approximately 9 × 4 × 1.5 cm (Figure 3) was identified and completely excised from the deep planes without intraoperative complications. A 1/8-inch Drenovac closed suction drain was placed and subsequently removed on the fifth postoperative day (Figure 4). The lesion was sent to pathology, who reported microscopic histopathology: The lesion is composed of dense fibrous connective tissue interspersed with mature adipose tissue. Thick, eosinophilic, fragmented, and disorganized elastic fibers were identified, presenting as elongated or globular structures compatible with elastofibroma (Figure 5); the patient recovered uneventfully.

## Discussion

Elastofibroma dorsi is an uncommon benign soft tissue tumor that predominantly presents in elderly women and young men. Its precise etiology remains unknown, with a reported global prevalence ranging from 2% to 2.73% on imaging studies and up to 11% to 24% in autopsy series, indicating that this condition is significantly underdiagnosed [6]. No specific epidemiological data for the Mexican population were identified in the available medical literature. Its most frequent anatomical site is the subscapular region, showing a clear unilateral predominance [2].

In our case, the patient presented with an elastofibroma dorsi, with histopathological confirmation showing fragmented and globular abnormal elastic fibers, which constitutes the hallmark feature of these neoplasms [7]. However, given its clinical presentation as a large mass fixed to deep planes, soft tissue sarcoma was considered a primary differential diagnosis, necessitating a definitive histopathological evaluation [4]. Furthermore, due to the limited resources at our institution, a soft-tissue ultrasound was performed instead of an MRI (the gold standard). This approach restricted the precise evaluation of deep fascial planes and adjacent tissue involvement, while also being operator-dependent and challenging for defining the intrinsic nature of the lesion [5].

Regarding therapeutic management, an excisional biopsy was deemed appropriate. Literature reviews indicate that surgical management in patients with symptoms yields excellent clinical outcomes and a favorable recovery [8]; however, detailed reports regarding the standard surgical technique for these neoplasms remain scarce. Additionally, a Drenovac closed-suction drain was placed to mitigate the risk of postoperative hematoma and seroma formation frequently reported in the literature [8]. This drain was removed on the fifth postoperative day during an outpatient visit without any complications, and subsequent follow-up revealed no adverse events. Notably, the surgical approach was performed with the patient in the prone position, which greatly facilitated the procedure given the anatomical location of the tumor.

Regarding the anesthetic management, it is worth noting that local anesthesia with 2% lidocaine combined with intravenous sedation (propofol and fentanyl) was utilized. This technique provided adequate pain control and modulation during both the perioperative and postoperative periods, despite the deep plane dissection. This strategy diverges from most published medical literature, which commonly recommends general anesthesia due to the extent of the lesion, depth of dissection, and optimal pain management requirements [3].

## Conclusions

Elastofibroma dorsi must be strongly considered as a differential diagnosis for soft tissue sarcomas. Furthermore, a thorough clinical history and physical examination must be coupled with appropriate imaging studies for correct classification and management. Currently, there is limited to no definitive evidence regarding the optimal standardized surgical approach. Consequently, further studies are necessary to establish appropriate management algorithms, taking into account the optimization of medical resources alongside comprehensive perioperative and postoperative care.
